# RETRACTED: Zhang et al. A Novel Framework for Reconstruction and Imaging of Target Scattering Centers via Wide-Angle Incidence in Radar Networks. *Sensors* 2025, *25*, 6802

**DOI:** 10.3390/s26134029

**Published:** 2026-06-25

**Authors:** Ge Zhang, Weimin Shi, Qilong Miao, Xiaofeng Shen

**Affiliations:** 1 School of Information and Communication Engineering, University of Electronic Science and Technology of China, Chengdu 611731, China; 2 School of Microelectronics and Communication Engineering, Chongqing University, Chongqing 400044, China

The Journal retracts the article titled “A Novel Framework for Reconstruction and Imaging of Target Scattering Centers via Wide-Angle Incidence in Radar Networks” [1], cited above.

Following publication, the authors contacted the Editorial Office to raise concerns regarding the inconsistent definition of the angular segmentation penalty factor and a mismatch between the probabilistic model and the parameter update scheme published in this article [1].

In accordance with the Journal’s standard procedures, the Editorial Office and Editorial Board conducted an investigation, which confirmed that in Equation (26), the conditional logic for the penalty factor contains a repetition error in the angular interval specification for the case where the angle lies outside the segment range. The current formulation leads to ambiguous execution logic in the EM iteration, which may affect the correctness of the non-coherent integration process. In addition, in Equation (21), the noise is modelled using a scalar variance, whereas in Equation (27), the noise power is updated on a per-node basis. This inconsistency between the assumed noise model and the estimation procedure undermines the theoretical consistency of the hyperparameter estimation in the EM step. These issues affect the reliability of the presented results and the reproducibility of the methodology. Consequently, the Editorial Office, Editorial Board, and authors have decided to retract this article in accordance with MDPI’s retraction policy.

This retraction was approved by the Editor-in-Chief of the *Sensors* journal.

The authors agreed to this retraction.
